# The legislative and regulatory framework governing herbal medicine use and practice in Kenya: a review

**DOI:** 10.11604/pamj.2017.28.232.12585

**Published:** 2017-11-15

**Authors:** Mitchel Otieno Okumu, Francis Okumu Ochola, Allan Odhiambo Onyango, James Mucunu Mbaria, Daniel Waweru Gakuya, Laetitia Wakonyu Kanja, Stephen Gitahi Kiama, Mary Atieno Onyango

**Affiliations:** 1 Department of Public Health, Pharmacology and Toxicology, Faculty of Veterinary Medicine, University of Nairobi, P.O BOX 29053-00625 Nairobi, Kenya; 2 Department of Pharmacology and Toxicology, Faculty of Medicine, Moi University, P.O BOX 3900-30100 Eldoret, Kenya; 3 Onyango Allan and Company Advocates, 3rd Floor, Pioneer House, P.O BOX 50485-00100 Nairobi, Kenya; 4 Department of Clinical Studies, Faculty of Veterinary Medicine, University of Nairobi, P.O BOX 29053-00625 Nairobi, Kenya; 5 Department of Veterinary Anatomy and Physiology, Faculty of Veterinary Medicine, University of Nairobi, P.O BOX 29053-00625 Nairobi, Kenya; 6 Department of Pharmacology, School of Medicine, Maseno University, P.O BOX Private bag, Maseno, Kenya

**Keywords:** Herbal medicine, legislation, regulatory framework, Kenya

## Abstract

Complementary and alternative medicine is an integral component of primary healthcare in Kenya. This is because the infrastructural health setup in the country is inadequate in catering for all the medical needs of the population. This particularly holds true in the rural areas where many rural folk rely on products of herbal origin to offset their healthcare needs. More often than not these products are an elaborate cacophony of several different substances of biological origin and thus need personnel adept in their preparation. Sadly, due to loopholes in legislation and regulation, quacks have a field day in the practice. Moreover, the process of planting, harvesting, preparation and storage of herbs and related products dictates that a significant number of people will ultimately be involved in the whole process. This is likely to set the stage for manipulation and compromise of the safety, quality and efficacy of these products. This state of affairs appears unabated especially in the context of the current legal and regulatory framework governing herbal medicine use and practice in Kenya. Not only are these laws inadequate, they are shrouded in ambiguity, open to interpretation and the authorities mandated to implement them often end up performing duplicate roles. The aim of this review is to critique the legal and regulatory provisions governing herbal medicine use and practice in Kenya. In conclusion, laws and regulations meant to control herbal medicine use and practice in Kenya are wanting. Clear and definitive legislation on herbal medicine use and practice coupled with effective implementation by mandated institutions will go a long way in inspiring confidence to all stakeholders of herbal medicine.

## Introduction

Controversy, doubt, suspicion and skepticism have courted the use and practice of herbal medicine since time immemorial [1-3]. However, if the ever expanding body of knowledge on claim validation and evidence based medicine is anything to go by, the situation seems to be improving. Moreover, emerging safety concerns arising from the use of allopathic/conventional drugs have served to improve the appeal of herbal medicine [4, 5]. In Kenya and by extension many developing countries, cultural and socio-economic factors coupled with the prevalence of a multitude of life-threatening disease conditions favour the use of herbal medicines [1, 6]. This is not to say that the use of herbal medicine is not popular in the developed world, far from it. Recent evidence suggests that herbal medicine use in the West is on the rise [5, 7]. In the local context, herbal medicine appears to many as a “cash-cow”. This allure has consequently prompted some locals and foreigners alike to venture into traditional medicine with the sole intention of making money [1]. Kenya is a developing nation with vast resources in herbs and folk knowledge supporting their use [8]. However, from a purely legal and regulatory standpoint, there seems to be glaring inadequacies in the mechanisms meant to control herbal medicines in the country. This article reviews the legal and regulatory framework of herbal medicine control in Kenya and highlights some of the challenges the legislation encounters and proposes recommendations for improving the existing framework.

## Methods

The present work is an in depth analysis of the legal and regulatory framework governing herbal medicine use and practice in Kenya. In this work we begin by giving an overview of herbal medicine practice in Kenya, diseases treated by herbal medicine practitioners, shortcomings of herbal medicine and ultimately critique the current regulatory and legislative framework governing herbal use and practice in the country by examining parliamentary laws, bills and policy documents that are herbal medicine oriented.

## Current status of knowledge

Kenya is a developing nation [9]. As such it is grappling with a shortage of skilled healthcare workers [10]. The situation is particularly dire in the rural areas. Practitioners of herbal medicine have thus found a niche' in the healthcare system in the country to the extent that they are recognized by the Ministry of Health [11]. However, the extent of this recognition remains largely vague and abstract. Studies on treatment seeking behaviour of Kenyan households reveals that practitioners of herbal medicine are often consulted when access to allopathic health workers is limited [12]. It is worth noting that the ratio of herbal practitioners to the general population is greater than the ratio of practitioners of allopathic/conventional medicine to the same population [13]. Thus, it is not surprising that in the grand scheme of things, a staggering 70% of the Kenyan populace rely on herbal medicine to offset their primary healthcare needs [14]. Furthermore, a recent survey of the demographic of herbal practitioners in Kenya established that more women are involved in the practice of herbal medicine than their male counterparts. The study also brought to light the existence of a simple scheme of specialization that defined different cadres within the practice of herbal medicine [15]. This is as depicted on Table 1; However, despite the huge dependence of rural folk on the services of practitioners of herbal medicine, practitioners of allopathic medicine as well as some members of the general public have a dim view of herbal medicine practice [16, 17]. The common train of thought is that these practitioners are dishonest in their undertakings with the public [14].

**Table 1:** Cadres of specialization within the practice of herbal medicine in Kenya

Specialization	Repertoire/skillset
Herbalist	Concoct medicines from plant parts as well as other materials. Treat respiratory, digestive and urinary conditions as well as intestinal parasites
Generalist	Use plant products but not considered specialists for any specific diseases
Dentists	Equipped with knowledge of particular plant products with tooth relieving properties. They also remove decayed/ broken teeth
Traditional Birth Attendants	Middle aged/older women with vast experience and knowledge in all herbs related to reproduction
Bone setters	Manage bone ailments, malformations using herbal medicine
Spiritual/faith healers	Relieve stress, depression, mental disturbances by use of herbal remedies

Adopted from the Health, Nutrition and Population (HNP) discussion paper [15]

There seems to be a consensus that these practitioners prey on a select proportion of the population: the less fortunate, the ignorant who have chosen to bury their heads in the sand as far as healthcare is concerned, those who may not actually be sufferers of any particular disease affliction or those likely to recover without any medical intervention [15]. However, on the backdrop of proven historical efficacy of some of the concoctions in the armamentarium of practitioners of herbal medicine, it may be foolhardy to rubbish some of the claims that have emerged from the practice [18, 19]. Thus, the relevance of practitioners of herbal medicine in offsetting primary healthcare needs of the population looms large [2, 20, 21]. Furthermore, the survival of the practice of herbal medicine appears to be buoyed by the fact that, in most cases practitioners of herbal medicine know their clientele on a personal level and have no red tape as far as payment is concerned; payment can be made in cash or kind [15]. The better part of the last two decades has seen many private organisations in Kenya venture into the field of herbal medicine [22]. In the same vein, many community oriented projects have been advanced with the objective of protecting local practices [23]. However, the mushrooming of many pharmacy outlets in various parts of the country and the increased tendency of members of the public to self-medicate threatens the livelihood of practitioners of herbal medicine [15].

**Diseases treated by practitioners of herbal medicine in Kenya**: There may be glaring inconsistencies in the methodology employed by practitioners of herbal medicine and that employed by practitioners of conventional medicine in diagnosing disease conditions [24, 25]. However, it could be argued that in most respects, the two forms of medicine aim to bring about therapeutic benefit to patients. Malaria, worm infestations, asthma, flu, pneumonia, arthritis, tuberculosis, cancer, tooth and stomach ache, diarrhoea, diabetes, ulcers and HIV are some of the diseases that are treated by practitioners of herbal medicine in Kenya [15, 26-29].

**Shortcomings of herbal practice**: As with many professions, the practice of herbal medicine is not without its limitations. It may be important to view the challenges of the practice of herbal medicine from the perspective of the practitioners of herbal medicine and from the perspective of the community:

**Practitioner's perspective**: The factors that influence the use of herbal medicine are diverse in their nature, differing from country to country [30]. However, dwindling plant resources due to urbanisation and population pressure, restrictive harvesting practices, non-payment of services offered, lack of acceptance/formal recognition by the relevant authorities, lack of representation, poor harvesting and storage conditions, lack of good will between practitioners of herbal medicine and those of allopathic medicine, religious beliefs, increased public access to health related information are some of the common impediments that have dogged the practice of herbal medicine as far as the practitioners of herbal medicine are concerned [31-33]. Misidentification of herbs, with potentially dire repercussions [34] and anxiety on the part of the practitioners of herbal medicine to divulge key tennets of their practice are also a challenge to the profession. Competition with foreign practitioners of herbal medicine has also stifled the environment under which practitioners of herbal medicine operate [1]. The practitioners are also concerned by the infiltration of quacks in this practice and dishonest researchers who acquire knowledge from them and use it for their own benefits.

**A community perspective**: The notion that herbal medicines are safe on the basis of their source (natural origin) may not always hold water [2]. In the recent past safety concerns have been on the rise [35]. The situation is further exacerbated by the concomitant use of allopathic medicine and herbal drugs [36, 37]. Moreover, many of the herbal medicines currently available on the market are devoid of any scientific evidence of safety and efficacy [38, 39]. Misinformation and outlandish claims on some herbal products is a major stumbling block as far as herbal medicine practice and use is concerned [40, 41]. The misguided belief that herbal medicines are a panacea has been spectacularly advanced to a varying degree of success by some proponents of herbal medicine use and practice [42]. Moreover, an increased demand for traditional medicine services may lead to some practitioners employing unscrupulous advertising practices to lure unsuspecting persons to their establishments [1, 43]. Some of the adverts are laced with attractive language and catchy phrases meant to capture the hearts and minds of their target audience [1, 44]. In addition, these practitioners refer to themselves by some carefully crafted often rhetorical title (s) meant to perpetuate authenticity in the message being conveyed [44]. Research into herbal medicine is labour, time and cost intensive. Vested interests by some researchers may lead them to harbour bias while reporting on the results of their research [45]. Contamination of herbal medicines by heavy metals is an emerging problem. This mainly occurs during the process of cultivation [46]. Deliberate adulteration is also practiced in the belief that the therapeutic effects of the herbal medicine will increase many fold [47, 48].

**Pharmacy and poisons act, cap 244, laws of Kenya**: This is an act of the parliament of Kenya that makes provision for the control of the profession of pharmacy and the trade in drugs and poisons [49]. The Act describes a *drug* as “any medicine, medicinal preparation or therapeutic substance” [50]. Furthermore, the Act defines a *medicinal substance* as “any medicine, substance, product or article that is claimed to be useful for any of the following purposes; a) treating, preventing or alleviating disease or symptoms of disease; b) diagnosing disease or ascertaining the existence, degree or extent of a psychological condition; c) preventing or interfering with the normal operation of a physiological function in human beings or animals, whether permanently or temporarily” [51]. Thus, from the foregoing it can be argued that if a particular herbal medicinal product is capable of performing some or all of the above, then it qualifies for regulation under this law. It is also instrumental to note that the Act also defines the term *manufacture* to mean “any process carried out in the course of making a product or medicinal substance and includes packaging, blending, mixing, assembling, distillation, processing, changing of form, application of any chemical or physical process in the preparation of a medicinal substance/product” [51]. In our opinion, in preparing herbal medicine all or some of these processes are applied and thus it may be safe to draw a conclusion that herbal medicine should be covered under this legislation. Additionally, the Act also stipulates that “no person shall sell by retail an article consisting of or comprising a substance recommended as a medicine unless the article itself is legibly labelled” [52]. The label should contain an appropriate description of the contents including the active constituents present as well as the quantitative particulars of the constituents or ingredient's [52].

In our opinion, the strict requirements highlighted above do not exclude herbal medicine. This then poses the question on how can herbal medicines be strictly in line with this Act considering the very nature of herbal medicine. The Act also has penal provisions and provides succinctly that" no person shall; a) manufacture any medicinal substance unless he has been granted a manufacturing license by the Pharmacy and Poisons Board (Kenya); b) each manufacturing license shall expire on the 31^st^ December of every year and the renewal shall be subject to compliance with conditions prescribed by the Board; c) no person shall manufacture any medicinal substance for sale unless he has applied for and obtained a license from the Board in respect of each substance intended to be manufactured; d) any person who intends to manufacture a medicinal substance shall make an application in the prescribed form for the licensing of the premises and the application shall be accompanied by the prescribed fee; e) medicinal substances under production in any manufacturing premises must comply to methods of manufacture approved by the Board; f) no person shall advertise any drug except with the written permission of the board [53]. Remarkably, practitioners of herbal medicine seem to have a blatant disregard for this law. However, before accusing practitioners of herbal medicine of having contravened the law, it is imperative to ask a fundamental question on how well the law is being implemented. On the other hand, feigning ignorance of the law or being unaware of its existence cannot be a basis upon which culpability of an offence is denied [54].

**Anti-doping act, laws of Kenya**: This is an act of parliament of Kenya that makes provision for the implementation of the United Nations Educational, Scientific and Cultural Organisation (UNESCO) convention against doping in sport. The ultimate aim of this Act is to protect the health of athletes through regulating sporting activities and ensuring that they are devoid of prohibited substances [55]. Section 42 (4) of the Anti-Doping Act provides that a medical practitioner, pharmacist, veterinary surgeon, dentist, nurse, physiotherapist, traditional herbalist or any other health professional who; a) prescribes or dispenses prohibited substances or methods to an athlete with the intent of doping; b) unlawfully administers prohibited substances or method to an athlete; c) acquires stocks or is found in unlawful possession of prohibited substances or; d) aids, abates or in any way encourages the unlawful use of prohibited substances commits an offence and shall be liable upon conviction to a fine of not less than 3 million Kenya shillings or to imprisonment for a term of not less than 3 years or to both such fine and imprisonment and shall have his or her professional license revoked for a period of not less than 1 year [55]. From the foregoing it is interesting to note that this Act criminalizes the use of performance enhancing drugs in sports (including herbs). Moreover, it could be suggested that the imposition of a huge penal fine and (or) imprisonment may serve to deter potential law breakers. Testament to the effectiveness of this law, a herbalist has recently been charged in a court of law after breaching several statutes of this law [56].

**Witchcraft act, Cap 67, laws of Kenya**: This is an act of the parliament of Kenya which provides that “any person who, of his pretended knowledge of so-called witchcraft, intends to injure, cause fear, annoyance or injury in mind, person or property to any person shall be guilty of an offence” [57]. This then begs the question whether witch medicine synonymous with the practice of herbal medicine. For a long time, herbal medicine has been associated with witchcraft and black magic. Thus most people have shunned it, instead wanting to associate with modern medicine [1, 58]. Without a clear regulatory framework to govern the practice of herbal medicine how will the practice of acceptable herbal medicine be distinguished from witchcraft [59]. By the very nature of the African society some genuine herbalists have been accused of engaging in witchcraft whereas it is not so; the Act [57] penalizing the blanket accusation of people engaging in herbal medicine has been of little help.

**Narcotic and psychotropic substances (control) act, cap 245 laws of Kenya**: This is an act of the parliament of Kenya that makes provision for the control, possession and trafficking in narcotic drugs and psychotropic substances as well as the cultivation of certain plants [60]. The following definitions in the Act hold relevance to this review; a) cannabis: flowering or fruiting tops of the cannabis plant excluding the seeds and leaves; b) coca bush: plant of any species of the genus erythroxylon from which cocaine can be extracted; c) coca leaves: leaves of the coca bush from which cocaine can be extracted either directly or by chemical transformation; d) prohibited plant: any plant specified in the Third Schedule; e) opium: includes raw opium, powdered opium and opium wholly or partially prepared for any use or purpose, whatever its content of morphine may be; f) opium poppy: the plant of the species papaver somniferum or all parts (except the seeds) of the opium poppy after harvesting whether in their original form or cut, crushed/powdered; g) medicinal opium :opium that has undergone processes necessary to adopt it for medicinal use; h) narcotic drug: any drug specified in the first schedule; i) psychotropic substance: any substance specified in the Second Schedule or anything that contains any substance contained in that schedule; j) cultivate: with reference to plants, this includes growing the plant, sowing/scattering the seeds produced by the plant, nurturing/tending/ harvesting flowers/fruits/leaves or seeds or the whole or any part of the plant; k) illicit traffic: in relation to narcotic drugs and psychotropic substances means the cultivation of any coca/cannabis/opium plant, any participation/involvement in production/possession/sale/ purchase/transportation or consumption of narcotic drugs and psychotropic substances; l) manufacture: with regard to narcotic drugs and psychotropic substances means the making of preparations (otherwise than in a pharmacy on a prescription) with or containing such drugs/substances; m) trafficking: means the importation, exportation, manufacture, buying, sale, giving, supplying, storing, administering, conveyance, delivery or distribution by any person to be a narcotic drug or psychotropic substance [60].

From the onset it can be argued that if a particular herbal medicine fits the description as defined by any of the definitions a-i, then it may be safe to draw the conclusion that the herb fits the criteria to be covered under this legislation. Additionally, the definitions k-m describe some of the practices that may be carried out by practitioners of herbal medicine in their day to day undertakings. In the event that one is found to have committed an offence, the penal provisions of this law provide for an imprisonment term of between 10-20 years, a fine of up to three times the market value of the narcotic drug/psychotropic substance or a monetary fine of between 2500 US $ and 10,000$ [61]. In our opinion, these are meant to dissuade potential offenders against breaking the law. Moreover, since this law provides elaborate definitions of narcotic drugs/psychotropic substances and their associated processes, it may be suggested that as far as regulation of herbal medicine (of a narcotic/psychotropic nature) is concerned, the law is sufficient. However, the same standards seem not to have been applied to other herbal medicines (i.e. those which are not of a narcotic/psychotropic nature).

**Industrial property act 2001, laws of Kenya**: This is an act of the parliament of Kenya that makes provision for the promotion of inventive and innovative activities, facilitates the acquisition of technology through granting and regulating patents, utility models and industrial designs and provides for the establishment, powers and functions of the Kenya Industrial Property Institute (KIPI) for purposes connected with the provisions mentioned above [62]. For the purposes of this review, the following definitions are noteworthy; a) innovation: refers to any utility/technovation model/industrial design or any other non-patentable creations/improvements that may be deemed as deserving specified industrial property rights; b) invention: means a new and useful art/process/machine/method of manufacture/composition of matter that is not obvious. It may also mean any new/useful improvement of the aforementioned which is not obvious and has the capacity to be used/applied in trade/industry an includes an alleged invention; c) utility model: any form/configuration/disposition of an element of some appliance/utensil/tool, electrical and electronic circuitry/instrument/handicraft mechanism or other object or any part of the same allowing a better/different functioning/use/manufacture of the subject matter or that gives utility/advantage/environmental benefit, saving/technical effect not available in Kenya before and includes micro-organisms/other self-replicable material/products of genetic resources/*herbal as well nutritional formulations which give new effects* [63]. At this juncture the law seems appropriate as far as the regulation of herbal medicine is concerned. However, Part III of this law, which elaborates on patents and patentability seems to present a dissenting opinion. Under this section, an invention is defined as a solution to a specific problem in the field of technology [64]. The law goes on to give details on what may be considered for patents. Strikingly, discoveries, scientific theories and mathematical models are not considered as inventions and are thus excluded from patent protection [64].

Additionally, methods of treatment of the human or animal body by surgery/therapy, as well as diagnostic methods are not patentable except products for use in any such method [64]. Thus, from the foregoing, this law seems to offer contradictory statements. On one hand, the definition of “innovation” may be construed as broad enough to include herbal medicine. Thus, the implication is that they are covered under this law. On the other hand, the law expressly excludes discoveries and scientific theories from patent protection. It may be argued that “theory” is a principle tenet upon which herbal medicine practice is founded. Evidence of the safety and efficacy of herbal medicine is more often than not based on history and personal experiences [19] which in our opinion are theoretical concepts. Moreover, new medicinal uses of herbs are being discovered every so often both locally and globally [65]. Thus, by excluding discoveries and scientific theories from patent protection, it is our opinion that the law does not seem to recognise the “fluid” and diverse nature of the practice of herbal medicine. Moreover, the law makes provision that “everything made available to the public anywhere in the world by means of written disclosure (including drawings and other illustrations) or by oral disclosure, exhibit or other non-written means shall be considered prior art and excluded from patent protection” [65]. From the standpoint of a researcher on herbal medicine, this law appears to limit the avenues through which the researcher can dissipate findings of his/her work. In our opinion, this is a draconian law and implies that the presentation of herbal medicine research work findings in a workshop, conference or publication in a journal automatically serves to disqualify the said researcher from seeking a patent for his/her innovation. This then poses the question on how the herbal medicine researchers are meant to communicate findings of their work. According to Rawat and Meena [66], publications are an important and useful tool of demonstrating academic prowess to peers and aims to improve the social standing of researchers and their institutions.

**The 1994 convention on biodiversity**: This is a policy document that was signed and ratified by Kenya with the aim of protecting the knowledge of herbal medicine held by its indigenous folk [32]. It expressly recognized the rights of communities over traditional knowledge. However, it did not give a clear direction on what prerogative was to remain with the state. Moreover, the Kenya Copyrights Board recently drafted a bill on the National Policy on Traditional Knowledge, Genetic Resources and Folklore [67]. The draft bill recommends a system that would seek to control the use of traditional know-how in local communities. However, a lack of consensus on the meaning/implication/definition of “community” makes the matter of solving claims involving multiple partnerships difficult [32].

**National policy on traditional medicine and medicinal plants**: This was drafted in 2005 and emphasized the need to take inventory of all the medicinal plants in the country [32]. It also sought to encourage the setting up of nurseries and herb gardens with the ultimate goal of bio-conservation and research [32]. Unfortunately, these recommendations are yet to be passed into law by the Kenyan parliament.

**National Health Bill, 2015**: It may not be all doom and gloom. Herbal medicine regulation may be about to get a reprieve. This may come in the form of the yet to be adopted National Health Bill, 2015. This bill aims to create a central system of health mandated to establish, co-ordinate and regulate the relationship between national and county governments on all matters related to health; services, providers, products and technologies [68]. This bill has been developed within the framework of the new constitutional dispensation of 2010 [32]. Notably, the following definitions of interest to this review are captured; a) alternative medicine: complementary medicine which is inclusive of health practices that are non-indigenous to the traditions of the country and non-integrated in the health care system; b) traditional medicine: knowledge, skills and practices based on theories, beliefs and experiences indigenous to different cultures used in maintenance of health as well as in the prevention, diagnosis and improvement or treatment of physical and mental illness [69]. Additionally, the bill aims to establish an oversight authority (Kenya Health Oversight Authority) to among other things maintain a register of all health workers, ensure execution of the individual mandates of respective regulatory bodies and; c) arbitrate in conflict /dispute resolution amongst councils and boards. Part V of the bill explicitly highlights the role of the regulatory authority on health products and technologies [70]. Part VIII of the bill is exclusively dedicated to traditional and alternative medicine. Here, policies that regulate the practice of herbal medicine, registration and licensure of practitioners of herbal medicine at national and county levels as well as documentation, mapping and standardization of the practice are articulated [68]. Hitherto the drafting of this bill, the registration of practitioners of herbal medicine was a prerogative of the Ministry of Gender, Sports, Culture and Social Services [71]. However, the lack of a specific criteria of registering and subsequent licensing of practitioners of herbal medicine by this Ministry has the implication that these practitioners are not held to the high standards of practice that allopathic medicine practitioners are held to. According to the national health bill, herbal medicine practitioners will be mandated to form a council of their members. Oversight of this council will be a prerogative of the Ministry of Health rather than the Ministry of Culture.

## Conclusion

In conclusion we opine that; i) Protection of intellectual property rights of traditional knowledge holders should be of principle concern to the government. The current policies governing intellectual property rights are inappropriate for the protection of herbal medicine and related resources as are the mechanisms for the protection, access to and benefit sharing arising from traditional knowledge and related resources; ii) Practitioners of herbal medicine should be trained on quality control, ethics and basic medical education; iii) Forums that bring together research scientists and herbal medicine practitioners should be promoted with the aim of fostering collaborative work between the scientists and practitioners of herbal medicine; iv) The current pharmacopoeia on herbal medicine developed by Kokwaro et al [72] should be updated.

### What is known about this topic

- Herbal medicine is an integral part of the healthcare system of many developing countries and by extension, Kenya;
- There is no shortage in laws and policies aimed at controlling herbal medicine in Kenya;
- Pertinent issues abound on the implementation of herbal medicine laws in Kenya.

### What this study adds

- Recommendations for creating an enabling environment for genuine practitioners of herbal medicine to thrive;
- Recommendations for improving the skillset of genuine practitioners of herbal medicine;
- Recommendations for the establishment and maintenance of well-defined protocols for data collection on herbal medicine practice and products.

## Competing interests

The authors declare no competing interests.
